# Case of Abscess of the Brain

**Published:** 1853-04

**Authors:** Thomas F. Cock


*Case of Abscess of the Brain.* By Thomas F. Cock, M. D.—James McDonald, aged 40, native of Ireland, laborer by occupation, was admitted to Bellevue Hospital, August 14th, 1851. A few days before admission, while at work in the sun, he was suddenly attacked with a severe pain in the forehead, having previously been in good health, though of intemperate habits. The pain had continued up to the time of admission without intermission. There was no corresponding constitutional disturbance, no acceleration of the pulse, no obtuseness of the intellect, no paralysis; the only abnormal symptom being the pain in the head. His physical appearance was that of a robust, healthy man. ℞.—Mag. sulphat. ℥j, blister to back of neck and Hoffman's anodyne ʒss, the first night.

August 15th.—The salts have purged and the blister vesicated; no improvement. Ordered cups to temples, and calomel with rhubarb, ä grs. x.

16th.—No improvement; ordered blister to each temple, pulv. antimonialis, grs. iij, ter in die, for two days.

18th.—Blister to forehead 4+3; at night tinct. valerian and Hoffman's anodyne, ä ʒi, also tart. ant. et potass. gr. ⅛ every two hours.

19th.—Pain somewhat relieved since administration of tartar emetic. This improvement, however, was only temporary. Patient walks about the ward and yard, com-

---

pressing his head with his hands; continues to eat and digest his food as if in perfect health.

September 1st.—Pain increased, apetite failing; patient sits by his bedside, his head clasped tightly by his hands, and resting on the bed. Ordered pulv. antimonialis. From this time patient's intellect became more and more obtuse: he ceased to complain of pain in the head; repeated questions put to him without returning any answer. His pulse diminished in frequency, falling to 55 per minute. He lingered in this condition several days, and expired at five o'clock, P. M., September 5th.

*Autopsy.*—On the anterior portion of the frontal bone was found a space two inches in extent by one in breadth, irregular in shape, denuded of its pericranium, the external table being also partly gone; extending from this diseased portion there is apparently a slight fissure. The part of the bone deprived of its pericranium is over the external portion of the orbit. There is no disease of the internal table; the membranes were considerably congested, the dura mater over the diseased portion of brain was thickened and adhered. The cerebral tissue beneath it was soft and diffluent. A portion of the brain over the posterior cornua of the right ventricle was softened, as also in a marked degree the anterior wall of the left ventricle and under surface of the fornix. Posteriorly to the diffluent portion above described is an abscess as large as a walnut, the walls of which are formed by a hardened reddish mass. The brain in its vicinity was pale and softened, other organs healthy.—*N. Y. Jour. Med.*

